# Correction: *Slowpoke* functions in circadian output cells to regulate rest:activity rhythms

**DOI:** 10.1371/journal.pone.0251678

**Published:** 2021-05-12

**Authors:** The PLOS ONE Staff

## Notice of Republication

This article was republished on April 22, 2021, to correct inaccurate formatting in the term “rest:activity” found in the title and throughout the article. The publisher apologizes for the errors introduced during the typesetting process. Please download this article again to view the correct version.

## Supporting information

S1 File Originally published, uncorrected article. (PDF)

S2 File Republished, corrected article. (PDF)
