# Supplementary figures and images for: Slowpoke functions in circadian output cells to regulate rest:activity rhythms
Source: PLoS One. 2021 Mar 25;16(3):e0249215. doi: 10.1371/journal.pone.0249215 (PMC7993846; doi:10.1371/journal.pone.0249215)

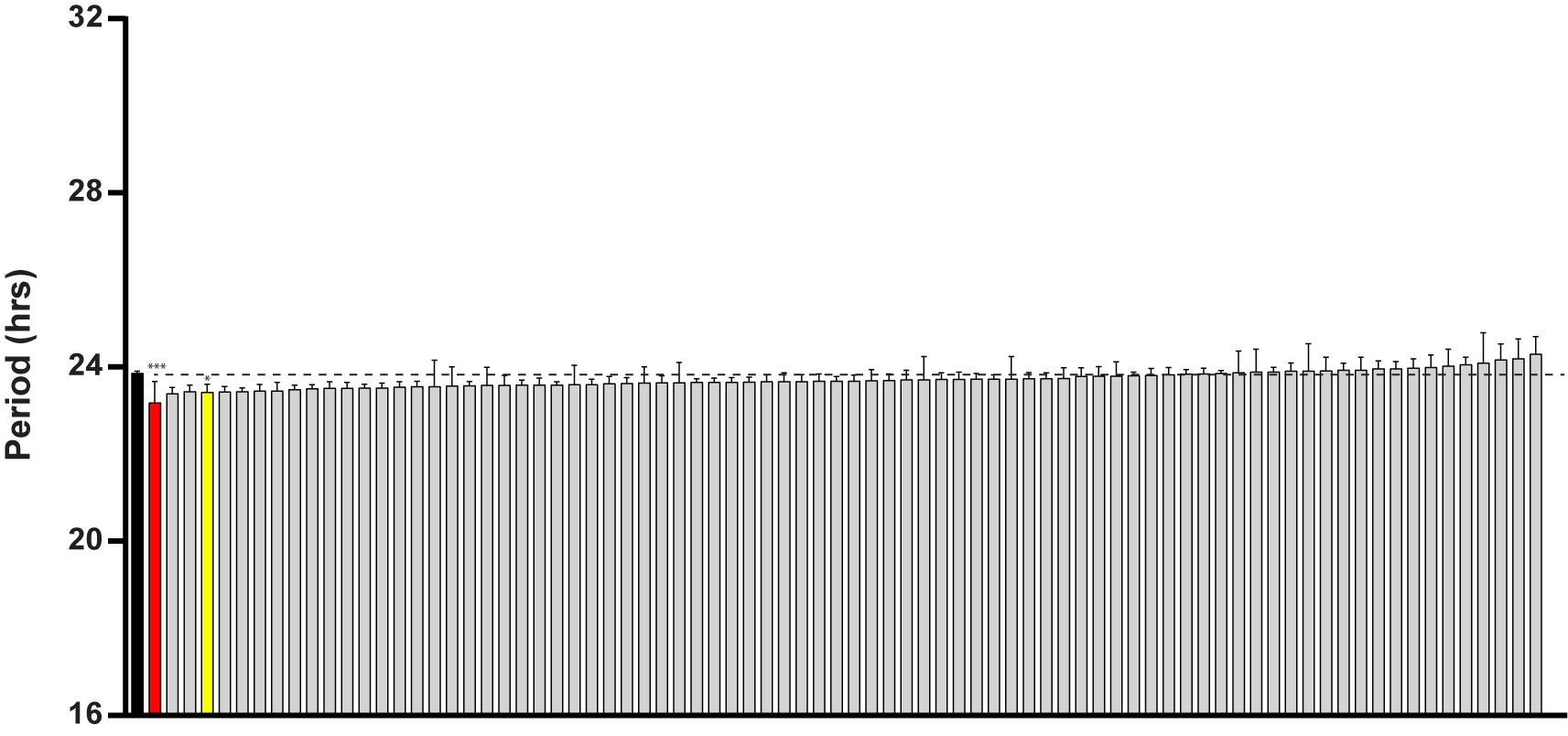

Supplement: S1 Fig — Screen results depict rest:activity rhythm period (mean ± 95% confidence interval) for all 80 experimental lines (in which SIFa/DH44-GAL4 was used to drive UAS-RNAi expression) as well as for GAL4 control flies (black bar). Only two lines—nAchRα3 RNAi1 (red bar) and EcR RNAi1 (yellow bar)—exhibited a statistically significant difference in period compared to control flies, and even in these cases, the effect sizes were small and inconsistent across other RNAi lines targeting these same genes. *p <0.05, ***p < 0.0001 compared to GAL4 control flies, Dunnett’s multiple comparisons test. (TIF) [file pone.0249215.s003.tif]
