# Supplementary material for: Slowpoke functions in circadian output cells to regulate rest:activity rhythms
Source: PLoS One. 2021 Mar 25;16(3):e0249215. doi: 10.1371/journal.pone.0249215 (PMC7993846; doi:10.1371/journal.pone.0249215)
Supplement: Attachment — Submitted filename: Response to Reviewers.docx [file pone.0249215.s005.docx]

**Response to Academic Editor:**

The revised version of the manuscript has improved considerably and all the reviewers issues have been addressed convincingly. However, I would still hold the authors to include a simple diagram/cartoon, as originally requested by Reviewer 1, that would help the non-specialist reader follow the Drosophila brain architecture and the experimental approach.

Based on the feedback of the academic editor (and initial input from Reviewer 1), we now include a diagram of the fly brain detailing the neurochemical classes of PI neurons. This has been added as Figure 1B. We hope that this aids the non-specialist reader to understand the Drosophila brain architecture and the specifics of our experimental approach.
